# Symptoms related to the visual system in migraine

**DOI:** 10.12688/f1000research.18768.1

**Published:** 2019-07-30

**Authors:** Robin M. van Dongen, Joost Haan

**Affiliations:** 1 Department of Neurology, Leiden University Medical Centre, Albinusdreef 2, 2333 ZA, Leiden, The Netherlands; 2 Department of Neurology, Alrijne Ziekenhuis, Simon Smitweg 1, 2353 GA, Leiderdorp, The Netherlands

**Keywords:** migraine aura, prolonged aura, persistent aura, retinal migraine, ophthalmoplegic migraine, photophobia, palinopsia, visual snow

## Abstract

Migraine is a common headache disorder characterized by often-severe headaches that may be preceded or accompanied by a variety of visual symptoms. Although a typical migraine aura is not difficult to diagnose, patients with migraine may report several other visual symptoms, such as prolonged or otherwise atypical auras, “visual blurring”, “retinal migraine”, “ophthalmoplegic migraine”, photophobia, palinopsia, and “visual snow”. Here, we provide a short overview of these symptoms and what is known about the relationship with migraine pathophysiology. For some symptoms, the association with migraine is still debated; for other symptoms, recent studies indicate that migraine mechanisms play a role.

## Introduction

Symptoms related to the visual system are common in migraine, a neurovascular brain disorder characterized by episodes of often-severe headache lasting between 4 hours and 3 days ^1^. In almost one third of patients with migraine, the headache is preceded by a visual aura ^2^ but there are several other visual symptoms related to migraine. The purpose of this narrative review is to provide a brief overview of these visual symptoms. Because migraine prevalence is high—lifetime prevalences are 33% in women and 13% in men ^2^—many physicians, especially general practitioners, neurologists, and ophthalmologists, will see patients with migraine. First, as background, the typical migraine aura is discussed, followed by the differential diagnosis of atypical auras. Next, visual symptoms other than migraine aura are reviewed: photophobia, palinopsia, and visual snow.

## Typical visual migraine aura

**Figure 1.**
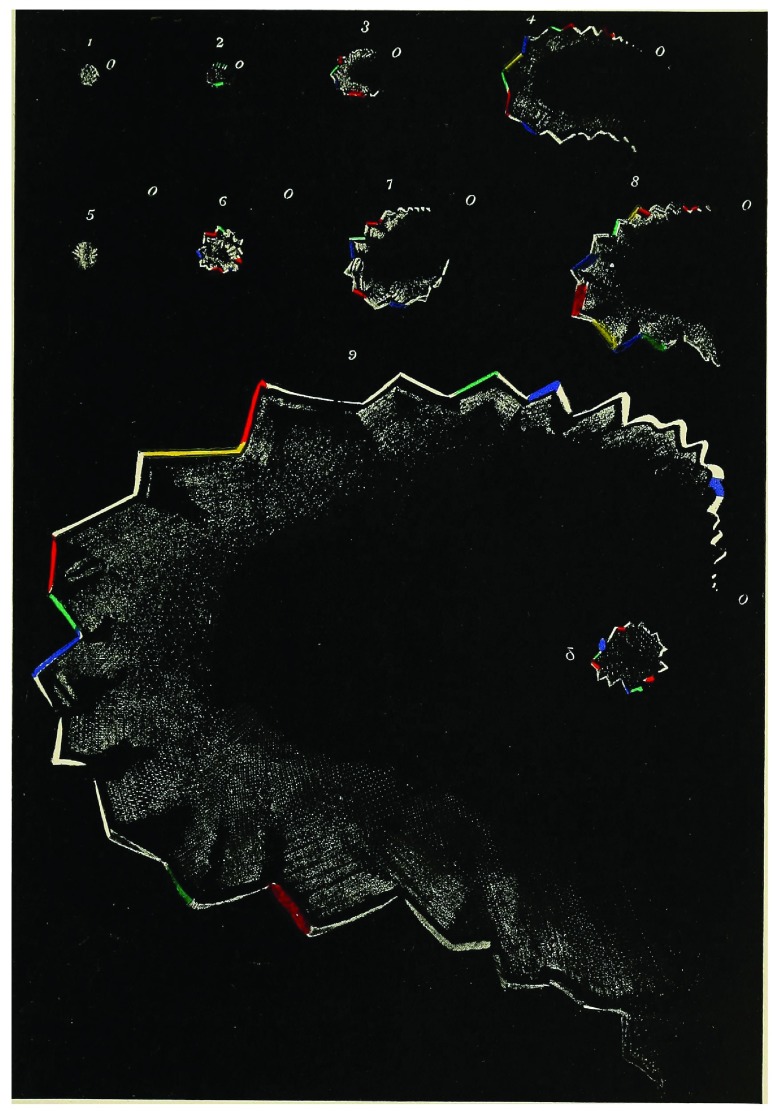
Example of a migraine aura. This picture illustrates the typical gradual expansion of a migraine aura along with the characteristic fortification spectrum.

In its most typical form, a visual aura begins with a “scintillating scotoma”, a small blind spot with a flickering, brightly colored, and typically jagged front—the so-called fortification spectrum—that generally expands in a C-shape to one side of the visual field ( Figure 1) ^1, 3^. The expansion is gradual and lasts between 5 and 60 minutes ^1^. These visual symptoms are fully reversible and should not be accounted for by another disorder, according to the third edition of the International Classification of Headache Disorders ^1^. The positive symptoms often take shapes other than a classic fortification spectrum. Patients commonly report that the aura starts with light flashes or balls of light that gradually expand across the visual field ^4, 5^. Furthermore, positive symptoms do not have to occur; patients can also report that the scotoma (that is, the blind spot or the hole in the visual field) has no colors or jagged lines. When patients close their eyes, the positive features of an aura remain visible. Besides having visual symptoms, patients may experience sensory symptoms, speech problems, and sometimes motor symptoms during their auras ^1^. If a person has had two attacks fulfilling criteria for migraine with aura, he or she is classified as a migraine with aura patient ^1^. If a person has only attacks without aura, he or she is classified as migraine-without-aura. Migraine with aura patients can still have attacks without aura. In a general-population study, median attack frequency was 12 attacks per year and 25% of patients with migraine had at least two attacks per month ^2^.

Gradual expansion is considered the most typical feature of a migraine aura ^1^. This corresponds with the suspected underlying pathophysiological mechanism called “cortical spreading depolarization” (CSD): a wave of intense neuronal and glial depolarization that is followed by neuronal depression. Preclinical and clinical studies suggest that the velocity of CSD through the occipital cortex is in line with the velocity of the visual spread observed by patients ^6– 8^. Patients can report a classic cascade in aura symptoms: the aura starts with visual symptoms and is followed by sensory symptoms, aphasia, and sometimes motor problems ^9^. It is believed that in these cases the CSD expands from the occipital cortex to the motor cortex ^10^. However, a prospective diary study showed that in 34% of patients the aura symptoms start simultaneously, suggesting that CSD may also start in a non-symptomatic brain area and reach two cortical areas at about the same time ^5, 11^. Sometimes patients report complex visual phenomena during their auras, such as distortions of observed body parts (for example, disproportionately large ears, sometimes referred to as the Alice in Wonderland syndrome) or visual splitting (one half of the observed face shifts upwards or downwards) ^3, 12, 13^. This suggests that more complex processes of visual perception, involving orientation and size, can also be altered during migraine attacks. Additionally, patients often report that during migraine auras their vision is “blurred” or “foggy” or that they see “heat waves”. In the diary study, this was registered in 33% of auras ^5^. However, information on gradual expansion was not collected. Furthermore, these symptoms have not been prospectively studied in migraine without aura patients. Therefore, there is still discussion about whether these symptoms are part of the aura spectrum.

In most patients, the aura is followed by a migraine headache. Typically, the headache starts after the aura has ended, although the headache can also start during or—in rare cases—before the aura ^5, 9, 14^. In a minority of attacks, the aura is not followed by headache ^4, 9^ and this classically occurs in elderly patients and is often referred to as “late-life migraine accompaniments” ^15^.

Current acute migraine medication is able to treat the headache only and has no effect on the auras. Preventive drugs such as beta-blockers, candesartan, topiramate, valproate, and amitriptyline and nortriptyline are generally considered when patients have an average of more than two attacks per month. When successful, the auras can also be prevented. For patients with frequent auras, lamotrigine can also be tried ^16^.

## Atypical visual migraine auras

Atypical auras are important to identify because of the differential diagnosis with other diseases. First, migraine auras can mimic stroke because of the homonymous visual field defects or additional focal symptoms ^17^. However, in migraine aura, symptoms gradually develop whereas in stroke they are often maximal from the start. Furthermore, classic aura features (colors and zig-zag lines) are generally not present in stroke ^18^. Nonetheless, it can be hard to discriminate between the two diagnoses and other clinical factors such as age and cardiovascular risk factors should be taken into account. When the aforementioned “late-life migraine accompaniments” in an elderly patient are considered, secondary causes such as cerebral amyloid angiopathy should also be excluded. Amyloid spells (that is, transient focal neurological episodes caused by superficial cortical siderosis) can mimic migraine auras ^19^.

Second, occipital epilepsy can also present with visual symptoms followed by headache but without the characteristic motor seizures. Classically, patients describe these symptoms as colored phosphenes ^3^, although phosphenes can also be reported as aura symptoms by patients with migraine ^20^. However, visual symptoms in epilepsy generally last shorter than 5 minutes and often start in the periphery of one temporal visual field whereas in migraine the auras generally start more to the center and expand to the periphery ^20^. These criteria are not specific and sometimes electro-encephalography recordings could help in diagnosing occipital seizures. The following entities have been described as variants of migraine aura, although the actual relationship with migraine has been criticized for some of them.

### Prolonged and persistent aura

In migraine, visual auras can last longer than the classic 60 minutes that is defined by the International Classification of Headache Disorders ^21, 22^. In one diary study, 26.4% of patients had a visual aura, a sensory aura, or speech problems that lasted longer than 60 minutes ^22^. This is often referred to as “prolonged aura”. However, visual auras lasting longer than 2 hours are considered rare and require additional investigation since they can be associated with cerebral infarction. If the aura is typical of previous auras (except in its duration) and there are imaging signs of ischemic infarction in the relevant brain area the term “migrainous infarction” is used to classify the infarct, but only if other causes of stroke are ruled out. Most cases of migrainous infarction are of cortical origin, suggesting that CSD could play a role; however, this could be confounding by indication since cortical infarcts are more likely to be classified as migrainous whereas subcortical infarcts may be attributed to a different cause ^23– 25^. There is still debate on whether increased prevalence of patent foramen ovale in patients with migraine could play a role as well ^23, 26^.

If infarction and other causes of binocular visual disturbances are excluded and aura symptoms last longer than 1 week, the term “persistent aura without infarction” is used ^1^. This is very rare. Symptoms can last months to years. Evidence on treatment is limited, lamotrigine is recommended on the basis of case series ^27^, and in one randomized controlled trial intranasal use of ketamine in patients with prolonged auras limited the aura severity but not the duration ^28^.

### Migraine aura status

Some patients with migraine with aura may experience a sudden and large increase in the frequency of their auras, typically without headache. If at least three auras occur over a period of 3 days, the term “migraine aura status” is used ^1^. However, this diagnosis requires that secondary causes, including occipital infarction, arterial dissection, reversible cerebral vasoconstriction syndrome, and posterior reversible encephalopathy syndrome, be excluded ^1, 29^. Retrospective studies indicate that a migraine aura status is rare ^30, 31^. Aura frequency can spontaneously normalize within a few weeks, but acetazolamide ^32^ or valproate ^31^ could aid in this process and prevent new episodes, although evidence is limited to case reports.

### Retinal migraine

The relevance of “retinal migraine” is still debated and unfortunately the term has been misused to describe visual auras, although these are binocular and homonymous and therefore of cortical origin. The term “retinal migraine” is reserved for monocular, fully reversible visual symptoms with at least two of the following criteria: the visual symptoms spread gradually during at least 5 minutes, last between 5 and 60 minutes, and are accompanied or followed within 60 minutes by headache ^1^. Importantly, secondary causes of monocular visual disturbances should be excluded. Critics of retinal migraine argue that symptoms are not monocular in patients with this diagnosis but binocular and that patients were not properly instructed to discriminate between monocular and binocular symptoms ^33^. Nonetheless, cases have been reported of recurrent, transient monocular visual disturbances followed by migraine headache in patients who were clearly instructed ^33^. Proponents additionally argue that CSD can occur in the retina, although these studies are still limited to *in vitro* models ^34^.

### Ophthalmoplegic migraine

Some patients with migraine—in particular, children with migraine—reported transient double vision after a migraine attack. This ophthalmoplegia involved mostly the third cranial nerve. Therefore, the term “ophthalmoplegic migraine” was introduced. A prerequisite is that the ophthalmoplegia is preceded by a “migraine-like” headache in the prior four days. However, a literature review of published cases showed that one third of cases did not have a headache fulfilling migraine criteria ^35^. Furthermore, the time between the headache and the ophthalmoplegia could be as long as 14 days and there was often focal enhancement of the third cranial nerve on magnetic resonance imaging. It was therefore re-classified as “recurrent painful ophthalmoplegic neuropathy”, and demyelization was suggested to play a role ^1, 35^.

## Photophobia

One of the main discriminating symptoms between migraine headache and other headache disorders is photophobia accompanying the headache, often occurring simultaneously with phonophobia ^1^. Patients often report that the migraine headache is worsened by the presence of light and that they have to lie in the dark.

Therefore, photophobia is classically viewed as an ictal symptom. However, studies focusing on the premonitory phase—most often defined as the 48 hours before the migraine attack starts—found that up to 49% of patients already experienced photophobia before the headache developed ^36, 37^. Furthermore, patients report that they have light aversion on days not preceding or following a migraine attack ^38^. There is increasing evidence for this hypersensitivity to light. Interictally, patients with migraine have a lower light discomfort threshold than controls ^39^. Additionally, many of them find patterns with high contrast more discomforting to look at ^38, 39^. This is supported by visual hyperexcitability studies using visual adaptation techniques ^40^, visual evoked potentials ^41^, and positron emission tomography (PET) brain imaging ^42^ in patients with interictal migraine. Interestingly, migraine with aura patients reported more discomfort than migraine without aura patients, suggesting that visual hyperexcitability could play a more prominent role in the former ^39^. This is in line with the hypothesis that migraine with aura patients have a lower threshold to experience a CSD. Although light stimulates nociceptive trigeminal neurons ^43^, it is thought that central processes involving thalamus and visual cortex have a more important role ^44^. There is also recent evidence from longitudinal studies that visual sensitivity already increases in the days preceding a migraine attack ^45, 46^. Although these discoveries are promising, they have not led to therapeutic options for photophobia.

## Palinopsia

**Figure 2.**
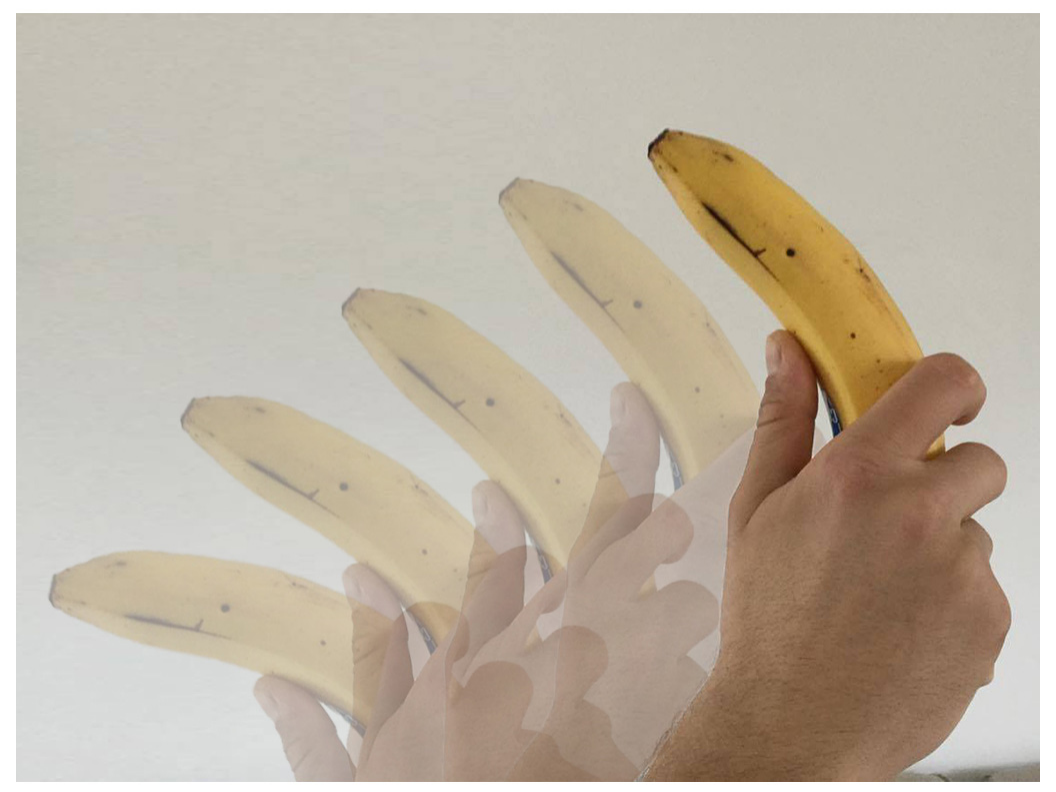
Example of palinopsia. An example of a moving object (the hand is moving from left to right) with perseverance of multiple images is shown.

Palinopsia is derived from the Greek words *palin* (again) and *opsis* (vision) and is used to describe perseveration of visual images. This can be experienced as seeing an afterimage of an object (after staring at an object and looking away) or as a series of images when an object is moving ( Figure 2) ^47^. Especially the former can be a normal physiological phenomenon when the stimulus was bright or there was a high contrast, and it results mostly in a negative afterimage: the afterimage is in complementary color. A positive afterimage, which has colors similar to those of the original image, is generally not considered physiological, although there is no strict cutoff in terms of frequency, duration, or other characteristics ^47^. There is evidence that patients with migraine are more prone to palinopsia, although the number of studies is limited and methods differ ^45, 48, 49^, complicating estimates on the prevalence of this symptom. In palinopsia, as in photophobia, it is thought that a central origin in the lateral geniculate nucleus of the posterior thalamus or visual cortex plays a role. Possibly altered sensory processing leading to palinopsia occurs primarily in the days preceding a migraine attack ^45^.

## Visual snow

**Figure 3.**
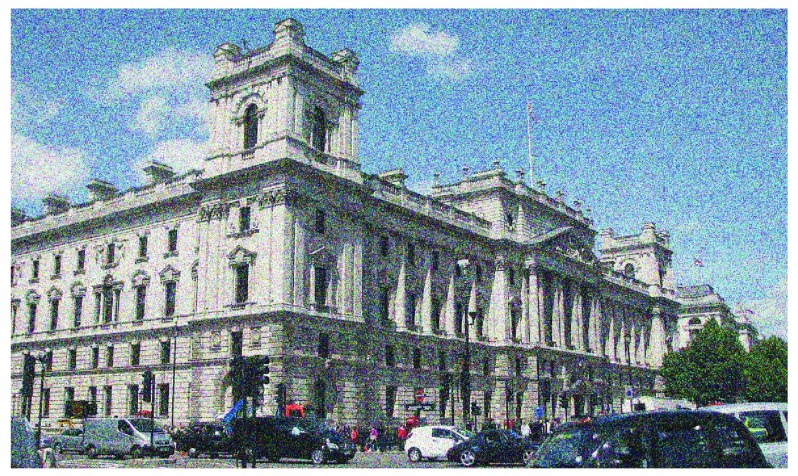
Example of visual snow. Patients with visual snow observe tiny dots in the entire visual field. The dots often resemble the “static” from a detuned analogue television because the dots “flicker on and off”. Classically, the dots are black/gray on a white background and white on a black background, but colors may vary.

Visual snow is characterized by the continuous presence of countless small dots in the entire visual field ( Figure 3) ^50^. Patients often describe it as “TV static from a detuned analogue television” since the dots are flickering on and off ^50^. Although the severity of the snow can vary during the day, symptoms are never fully absent. Visual snow was not considered a separate entity until 2014 ^50^. Earlier reports often referred to the snow as a form of “persistent migraine aura”, possibly because many patients have a history of migraine with aura ^27, 51– 55^. However, in visual snow, the classic migraine features such as scintillating scotomas and fortification spectra are absent ^1, 50, 54^. Furthermore, persistent migraine auras often start unilaterally and expand whereas patients with visual snow generally report that it started in the entire visual field and there was no spatial expansion ^50^.

Little is known about the epidemiology of visual snow ^50, 56^. Age of onset is often in the early twenties. In visual snow, in contrast to migraine, current data do not show an increased prevalence in females. Visual snow seems to be related to migraine: two case series found that between 47 and 59% had a history of migraine and that the prevalence of migraine with aura was relatively high compared with that of migraine without aura ^50, 56^. Furthermore, patients with migraine sometimes also report seeing visual snow, albeit transient and not continuous. In a prospective diary study, 8% of patients with migraine reported visual snow during their visual aura but not outside the aura ^5^.

A diagnosis of visual snow is made after exclusion of secondary causes of pan-field visual disturbances, such as lesions in the visual pathways and retina. Nevertheless, ophthalmic and neurological examinations in patients with visual snow are generally normal ^50^. Most patients report additional visual symptoms: palinopsia, enhanced entopic phenomena (excessive floaters or blue field entoptic phenomena and spontaneous photopsia), photophobia, and nyctalopia. Therefore, it was proposed that visual snow is part of a clinical syndrome ^50^. The syndrome criteria were proposed for research purposes and have no current clinical consequence for diagnosis or prognosis. Visual snow has also been reported as a persistent visual effect after intake of illicit hallucinogenic drugs such as LSD and ecstasy ^57^. However, since there are patients with visual snow who never used hallucinogenic drugs ^50^ and since visual snow is also reported in children ^58^, it seems that hallucinogenic drug use is not the only potential risk factor.

It is hypothesized that cortical hyperexcitability plays a role in visual snow as well. Theoretically, the visual disturbances can also be localized to bilateral retinal pathology; however, this seems unlikely since ophthalmological examinations and electro-retinograms are generally normal in patients with visual snow ^50^. Therefore, visual snow is generally considered a cortical problem. Indeed, there is some evidence that cortical excitability parameters are increased in patients with visual snow and without comorbid migraine. This has been tested by using visual tasks ^59^ and visual-evoked potentials ^60^, but findings still have to be replicated in larger studies. One study using [ ^18^F]-2-fluoro-2-de-oxy-D-glucose PET showed hypermetabolism in the lingual gyrus, an area that modulates visual processing, in visual snow patients compared with healthy controls ^61^. The same area was shown to be involved in previous migraine studies on photophobia ^42, 62^. However, because 14 out of 17 patients with visual snow also had comorbid migraine, it remains unknown whether this hypermetabolism is specific for visual snow ^61^. Treatment of visual snow is limited to case reports and expert opinion. Lamotrigine may sometimes help ^63, 64^.

## Conclusions

The visual aura is the most typical form of visual disturbances in migraine but there are several other visual symptoms that people with migraine report. In this review, we have given a short clinical overview of these symptoms and what is known about the relationship with migraine pathophysiology. Although there are currently no specific therapeutic options focusing on visual symptoms, recent studies have begun to unravel some of the mechanisms that are involved.

## References

[1] Headache Classification Committee of the International Headache Society (IHS) The International Classification of Headache Disorders, 3rd edition. *Cephalalgia.* 2018;38(1):1–211. 10.1177/0333102417738202 29368949

[2] Launer LJ Terwindt GM Ferrari MD: The prevalence and characteristics of migraine in a population-based cohort: The GEM study. *Neurology.* 1999;53(3):537–42. 10.1212/WNL.53.3.537 10449117

[3] Schott GD: Exploring the visual hallucinations of migraine aura: The tacit contribution of illustration. *Brain.* 2007;130(Pt 6):1690–703. 10.1093/brain/awl348 17264093

[4] Hansen JM Goadsby PJ Charles AC: Variability of clinical features in attacks of migraine with aura. *Cephalalgia.* 2016;36(3):216–24. 10.1177/0333102415584601 25944814

[5] Viana M Sances G Linde M: Clinical features of migraine aura: Results from a prospective diary-aided study. *Cephalalgia.* 2017;37(10):979–89. 10.1177/0333102416657147 27573009

[6] Leão AAP: Spreading depression of activity in the cerebral cortex. *J Neurophysiol.* 1944;7:359–90. 10.1152/jn.1944.7.6.359 20268874

[7] Hadjikhani N Sanchez Del Rio M Wu O: Mechanisms of migraine aura revealed by functional MRI in human visual cortex. *Proc Natl Acad Sci U S A.* 2001;98(8):4687–92. 10.1073/pnas.071582498 11287655 PMC31895

[8] Hansen JM Baca SM VanValkenburgh P: Distinctive anatomical and physiological features of migraine aura revealed by 18 years of recording. *Brain.* 2013;136(Pt 12):3589–95. 10.1093/brain/awt309 24264937

[9] Russell MB Olesen J: A nosographic analysis of the migraine aura in a general population. *Brain.* 1996;119(Pt 2):355–61. 10.1093/brain/119.2.355 8800932

[10] Ferrari MD Klever RR Terwindt GM: Migraine pathophysiology: lessons from mouse models and human genetics. *Lancet Neurol.* 2015;14(1):65–80. 10.1016/S1474-4422(14)70220-0 25496898

[11] Viana M Linde M Sances G: Migraine aura symptoms: Duration, succession and temporal relationship to headache. *Cephalalgia.* 2016;36(5):413–21. 10.1177/0333102415593089 26156076

[12] Kew J Wright A Halligan PW: Somesthetic aura: The experience of “Alice in Wonderland”. *The Lancet.* 1998;351(9120):1934. 10.1016/S0140-6736(05)78619-0 9654271

[13] Jürgens TP Schulte LH May A: Migraine trait symptoms in migraine with and without aura. *Neurology.* 2014;82(16):1416–24. 10.1212/WNL.0000000000000337 24658932

[14] Hansen JM Lipton RB Dodick DW: Migraine headache is present in the aura phase: A prospective study. *Neurology.* 2012;79(20):2044–9. 10.1212/WNL.0b013e3182749eed 23115208 PMC3511920

[15] Vongvaivanich K Lertakyamanee P Silberstein SD: Late-life migraine accompaniments: A narrative review. *Cephalalgia.* 2014;35(10):894–911. 10.1177/0333102414560635 25505036

[16] Lampl C Katsarava Z Diener H-C: Lamotrigine reduces migraine aura and migraine attacks in patients with migraine with aura. *J Neurol Neurosurg Psychiatr.* 2005;76(12):1730–2. 10.1136/jnnp.2005.063750 16291905 PMC1739463

[17] Nadarajan V Perry RJ Johnson J: Transient ischaemic attacks: Mimics and chameleons. *Pract Neurol.* 2014;14(1):23–31. 10.1136/practneurol-2013-000782 24453269 PMC3913122

[18] Lavallée PC Cabrejo L Labreuche J: Spectrum of transient visual symptoms in a transient ischemic attack cohort. *Stroke.* 2013;44(12):3312–7. 10.1161/STROKEAHA.113.002420 24178913

[19] Charidimou A Peeters A Fox Z: Spectrum of transient focal neurological episodes in cerebral amyloid angiopathy: Multicentre magnetic resonance imaging cohort study and meta-analysis. *Stroke.* 2012;43(9):2324–30. 10.1161/STROKEAHA.112.657759 22798323

[20] Hartl E Gonzalez-Victores JA Rémi J: Visual Auras in Epilepsy and Migraine - An Analysis of Clinical Characteristics. *Headache.* 2017;57(6):908–16. 10.1111/head.13113 28488762

[21] Viana M Sprenger T Andelova M: The typical duration of migraine aura: A systematic review. *Cephalalgia.* 2013;33(7):483–90. 10.1177/0333102413479834 23475294

[22] Viana M Sances G Linde M: Prolonged migraine aura: New insights from a prospective diary-aided study. *J Headache Pain.* 2018;19(1):77. 10.1186/s10194-018-0910-y 30171359 PMC6119171

[23] Wolf ME Szabo K Griebe M: Clinical and MRI characteristics of acute migrainous infarction. *Neurology.* 2011;76(22):1911–7. 10.1212/WNL.0b013e31821d74d5 21624990

[24] Laurell K Artto V Bendtsen L: Migrainous infarction: A Nordic multicenter study. *Eur J Neurol.* 2011;18(10):1220–6. 10.1111/j.1468-1331.2011.03364.x 21414105

[25] Lee MJ Lee C Chung C-S: The Migraine-Stroke Connection. *J Stroke.* 2016;18(2):146–56. 10.5853/jos.2015.01683 27283278 PMC4901947

[26] Koppen H Palm-Meinders IH Mess WH: Systemic right-to-left shunts, ischemic brain lesions, and persistent migraine activity. *Neurology.* 2016;86(18):1668–75. 10.1212/WNL.0000000000002538 26968513 PMC4854588

[27] Thissen S Vos IG Schreuder TH: Persistent Migraine Aura: New Cases, a Literature Review, and Ideas About Pathophysiology. *Headache.* 2014;54(8):1290–309. 10.1111/head.12392 24863140

[28] Afridi SK Giffin NJ Kaube H: A randomized controlled trial of intranasal ketamine in migraine with prolonged aura. *Neurology.* 2013;80(7):642–7. 10.1212/WNL.0b013e3182824e66 23365053

[29] Terrin A Mainardi F Maggioni F: The pathological spectrum behind migraine aura status: A case series. *Neurol Sci.* 2019;40(4):861–4. 10.1007/s10072-018-3671-0 30523547

[30] Beltramone M Donnet A: Status migrainosus and migraine aura status in a French tertiary-care center: An 11-year retrospective analysis. *Cephalalgia.* 2014;34(8):633–7. 10.1177/0333102414520763 24504530

[31] João AA Goucha TB Martins IP: Aura status: A not so frequent aura. *Cephalalgia.* 2014;34(14):1150–62. 10.1177/0333102414530525 24740515

[32] Haan J Sluis P Sluis LH: Acetazolamide treatment for migraine aura status. *Neurology.* 2000;55(10):1588–9. 10.1212/wnl.55.10.1588 11094126

[33] Hill DL Daroff RB Ducros A: Most cases labeled as "retinal migraine" are not migraine. *J Neuroophthalmol.* 2007;27(1):3–8. 10.1097/WNO.0b013e3180335222 17414865

[34] Wang M Chazot PL Ali S: Effects of NMDA receptor antagonists with different subtype selectivities on retinal spreading depression. *Br J Pharmacol.* 2012;165(1):235–44. 10.1111/j.1476-5381.2011.01553.x 21699507 PMC3252980

[35] Gelfand AA Gelfand JM Prabakhar P: Ophthalmoplegic "migraine" or recurrent ophthalmoplegic cranial neuropathy: new cases and a systematic review. *J Child Neurol.* 2012;27(6):759–66. 10.1177/0883073811426502 22241707 PMC3562350

[36] Maniyar FH Sprenger T Monteith T: The premonitory phase of migraine--what can we learn from it? *Headache.* 2015;55(5):609–20. 10.1111/head.12572 25919990

[37] Giffin NJ Ruggiero L Lipton RB: Premonitory symptoms in migraine: an electronic diary study. *Neurology.* 2003;60(6):935–40. 10.1212/01.wnl.0000052998.58526.a9 12654956

[38] Mulleners WM Aurora SK Chronicle EP: Self-reported photophobic symptoms in migraineurs and controls are reliable and predict diagnostic category accurately. *Headache.* 2001;41(1):31–9. 10.1046/j.1526-4610.2001.111006031.x 11168601

[39] Perenboom MJL Zamanipoor Najafabadi AH Zielman R: Quantifying visual allodynia across migraine subtypes: the Leiden Visual Sensitivity Scale. *Pain.* 2018;159(11):2375–82. 10.1097/j.pain.0000000000001343 30015708 PMC6203424

[40] Shepherd AJ: Increased visual after-effects following pattern adaptation in migraine: a lack of intracortical excitation? *Brain.* 2001;124(Pt 11):2310–8. 10.1093/brain/124.11.2310 11673331

[41] Chen WT Wang SJ Fuh JL: Persistent ictal-like visual cortical excitability in chronic migraine. *Pain.* 2011;152(2):254–8. 10.1016/j.pain.2010.08.047 21145169

[42] Boulloche N Denuelle M Payoux P: Photophobia in migraine: an interictal PET study of cortical hyperexcitability and its modulation by pain. *J Neurol Neurosurg Psychiatr.* 2010;81(9):978–84. 10.1136/jnnp.2009.190223 20595138

[43] Okamoto K Tashiro A Chang Z: Bright light activates a trigeminal nociceptive pathway. *Pain.* 2010;149(2):235–42. 10.1016/j.pain.2010.02.004 20206444 PMC2860692

[44] Goadsby PJ Holland PR Martins-Oliveira M: Pathophysiology of Migraine: A Disorder of Sensory Processing. *Physiol Rev.* 2017;97(2):553–622. 10.1152/physrev.00034.2015 28179394 PMC5539409

[45] Luedtke K Schulte LH May A: Visual processing in migraineurs depends on the migraine cycle. *Ann Neurol.* 2019;85(2):280–3. 10.1002/ana.25398 30556164

[46] McKendrick AM Chan YM Vingrys AJ: Daily vision testing can expose the prodromal phase of migraine. *Cephalalgia.* 2018;38(9):1575–84. 10.1177/0333102417741130 29110502

[47] Gersztenkorn D Lee AG: Palinopsia revamped: a systematic review of the literature. *Surv Ophthalmol.* 2015;60(1):1–35. 10.1016/j.survophthal.2014.06.003 25113609

[48] Belcastro V Cupini LM Corbelli I: Palinopsia in patients with migraine: a case-control study. *Cephalalgia.* 2011;31(9):999–1004. 10.1177/0333102411410083 21628437

[49] Kalita J Uniyal R Bhoi SK: Is palinopsia in migraineurs an enhanced physiological phenomenon? *Cephalalgia.* 2016;36(13):1248–56. 10.1177/0333102415625610 26767828

[50] Schankin CJ Maniyar FH Digre KB: 'Visual snow' - a disorder distinct from persistent migraine aura. *Brain.* 2014;137(Pt 5):1419–28. 10.1093/brain/awu050 24645145

[51] Liu GT Schatz NJ Galetta SL: Persistent positive visual phenomena in migraine. *Neurology.* 1995;45(4):664–8. 10.1212/wnl.45.4.664 7723952

[52] Rothrock JF: Successful treatment of persistent migraine aura with divalproex sodium. *Neurology.* 1997;48(1):261–2. 10.1212/wnl.48.1.261 9008529

[53] Jäger HR Giffin NJ Goadsby PJ: Diffusion- and perfusion-weighted MR imaging in persistent migrainous visual disturbances. *Cephalalgia.* 2005;25(5):323–32. 10.1111/j.1468-2982.2004.00858.x 15839846

[54] Wang YF Fuh JL Chen WT: The visual aura rating scale as an outcome predictor for persistent visual aura without infarction. *Cephalalgia.* 2008;28(12):1298–304. 10.1111/j.1468-2982.2008.01679.x 18727635

[55] Chen WT Lin YY Fuh JL: Sustained visual cortex hyperexcitability in migraine with persistent visual aura. *Brain.* 2011;134(Pt 8):2387–95. 10.1093/brain/awr157 21729907

[56] Lauschke JL Plant GT Fraser CL: Visual snow: A thalamocortical dysrhythmia of the visual pathway? *J Clin Neurosci.* 2016;28:123–7. 10.1016/j.jocn.2015.12.001 26791474

[57] Litjens RP Brunt TM Alderliefste GJ: Hallucinogen persisting perception disorder and the serotonergic system: a comprehensive review including new MDMA-related clinical cases. *Eur Neuropsychopharmacol.* 2014;24(8):1309–23. 10.1016/j.euroneuro.2014.05.008 24933532

[58] Simpson JC Goadsby PJ Prabhakar P: Positive persistent visual symptoms (visual snow) presenting as a migraine variant in a 12-year-old girl. *Pediatr Neurol.* 2013;49(5):361–3. 10.1016/j.pediatrneurol.2013.07.005 23968568

[59] McKendrick AM Chan YM Tien M: Behavioral measures of cortical hyperexcitability assessed in people who experience visual snow. *Neurology.* 2017;88(13):1243–9. 10.1212/WNL.0000000000003784 28251918

[60] Eren O Rauschel V Ruscheweyh R: Evidence of dysfunction in the visual association cortex in visual snow syndrome. *Ann Neurol.* 2018;84(6):946–9. 10.1002/ana.25372 30383334

[61] Schankin CJ Maniyar FH Sprenger T: The relation between migraine, typical migraine aura and "visual snow". *Headache.* 2014;54(6):957–66. 10.1111/head.12378 24816400

[62] Denuelle M Boulloche N Payoux P: A PET study of photophobia during spontaneous migraine attacks. *Neurology.* 2011;76(3):213–8. 10.1212/WNL.0b013e3182074a57 21148120

[63] Unal-Cevik I Yildiz FG: Visual Snow in Migraine With Aura: Further Characterization by Brain Imaging, Electrophysiology, and Treatment--Case Report. *Headache.* 2015;55(10):1436–41. 10.1111/head.12628 26307008

[64] White OB Clough M McKendrick AM: Visual Snow: Visual Misperception. *J Neuroophthalmol.* 2018;38(4):514–21. 30095537 10.1097/WNO.0000000000000702

